# Pterin-Based Red Coloration Predicts the Outcome of Male–Male Competition in Guinan Toad-Headed Lizard

**DOI:** 10.3390/ani14202923

**Published:** 2024-10-11

**Authors:** Xiao Xiao, Song Tan, Kehu He, Ying Chen, Lin Cui, Bicheng Zhu, Xia Qiu, Yin Qi, Weizhao Yang

**Affiliations:** 1 Chengdu Institute of Biology, Chinese Academy of Sciences, Chengdu 610041, China; xiaoxiao1@cib.ac.cn (X.X.); tansong@cib.ac.cn (S.T.); chenying@cib.ac.cn (Y.C.); cuilin@cib.ac.cn (L.C.); zhubc@cib.ac.cn (B.Z.); 2 University of Chinese Academy of Sciences, Beijing 100049, China; 3 Forestry Station of Guinan County, Guinan 813199, China; hekehu2024@126.com; 4 College of Life Sciences, China Jiliang University, Hangzhou 310018, China; qiu999@cjlu.edu.cn

**Keywords:** animal coloration, pterins, *Phrynocephalus guinanensis*, honest signaling, intrasexual competition

## Abstract

**Simple Summary:**

The study of animal coloration has long been a central theme in biological science. Bright colors that often derived from pigments serve as vital social signals across species, conveying information about individual quality, social status, and reproductive fitness. Pterins as a group of endogenous pigments capable of producing bright colors, have received relatively little attention so far. Whether pterin-based colors represent signals of individual quality remains unclear. The vibrant red coloration displayed by male Guinan toad-headed lizards offers an ideal model to investigate this question. Through an integrated approach combining metabolomics, colorimetry, and behavioral experiments, we found that this red coloration was not correlated with body size, bite force, or testosterone level, and it did not influence female mate choice. However, red intensity predicted male–male competition outcomes, with deeper red males more likely to win contests. Our findings suggest that pterin-based coloration can serve as a signal of male quality. This study provides novel insights into the role of pterin-based colors in animals.

**Abstract:**

Animal coloration offers a unique opportunity to explore the evolutionary mechanisms underlying phenotypic diversity. Conspicuous coloration caused by pigments plays a crucial role in social signaling across multiple species by conveying information about individual quality, social ranks, or reproductive condition. Nevertheless, most previous studies have focused predominantly on colors produced by the exogenous pigments—carotenoids. Pterins are another prevalent group of conspicuous pigments, which can be produced endogenously and have received comparatively little attention. Whether pterin-based colors represent reliable signals remains elusive. The remarkable red ventrolateral coloration exhibited by males of the Guinan toad-headed lizard (*Phrynocephalus guinanensis*) in the Mugetan Desert presents an ideal model for investigating pterin-based coloration. Through electron microscopy and metabolomic identification, we discovered three types of pterin pigments within xanthophores. Integrating a series of morphological measurements and behavioral experiments, we found that this red coloration was not correlated with body size, bite force, and testosterone level, nor did females show a preference bias toward it. However, the red intensity predicted male–male competition outcomes, with deeper red males being more likely to emerge as winners. Our results indicated that the pterin-based coloration could convey information about male quality, suggesting its potential role in honest signaling, given the vital importance of pterin metabolism in physiological processes. This study provides a novel case into the understanding of pterin-based colors in animals.

## 1. Introduction

The study of animal coloration has been a long-standing topic within biological science since the 19th century [1,2,3]. Coloration serves multiple functions such as crypsis, intraspecific communication, anti-predator signaling, and thermoregulation [4,5]. In recent decades, technological advances, including digital imaging, spectrophotometry, and visual modeling, have enabled the precise quantification of individual coloration [3]. These progress, in conjunction with other -omics approaches, have facilitated a deeper understanding of how colors are formed and maintained [6,7,8]. Consequently, animal coloration offers a unique lens through which to explore the evolutionary mechanisms underlying phenotypic diversity.

Conspicuous pigmentary colors play a crucial role in social signaling in various species [4]. Animal coloration can be generated by either nanostructure or pigments [5,9]. Pigmentary coloration arises from small molecule compounds within xanthophores (top layer of dermis) and melanophores (bottom layer) that selectively absorb specific wavelengths of light. This absorption interacts with iridophores (middle layer), which causes light of other wavelengths to be reflected, creating the visible colors [10]. The most common pigments include carotenoids, melanins, pterins, and ommochromes [11,12,13]. Nevertheless, the majority of previous studies have focused predominantly on the former two pigments, particularly carotenoids. As a well-studied group of pigments, carotenoids often cause conspicuous red or yellow coloration in animals, which can only be gained through dietary intake rather than self-synthesis [13]. Consequently, in resource-limited environments, a brighter color may signal an individual’s dominant state, potentially correlating with larger body size, advantage over same-sex rivals in resource competition or increased attractiveness to potential mates [14]. This “honest signaling hypothesis” has been observed in multiple species, such as house finch [15] (*Carpodacus mexicanus*), cichlid fishes [16] (*Tropheus duboisi*), and orangethroat darter [17] (*Etheostoma spectabile*). In contrast, pterins, as another common group of pigments, have received very limited attention [18]. Although they also frequently display bright coloration, pterins can be synthesized endogenously by animals, which is unlike carotenoids [11,19,20]. Pterins have been identified in multiple taxa, including insects, fishes, amphibians, reptiles, and mammals [21,22,23,24,25]. However, whether pterin pigments can serve as signals remains unclear. The investigation of pterin-based coloration may provide valuable insights into the questions of how biological colors arise and function.

**Figure 1:**
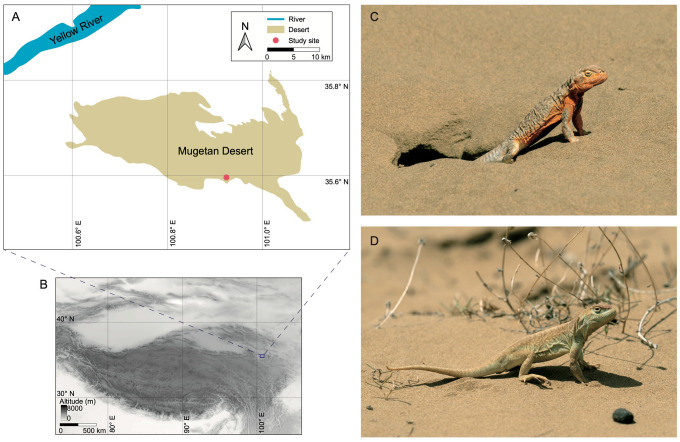
Study site and photographs of *Phrynocephalus guinanensis*. (**A**) The location of the sampling and field work site in Mugetan Desert. (**B**) The location of the Mugetan region in the Qinghai–Tibetan Plateau. (**C**) Photograph of a male *P. guinanensis*. (**D**) Photograph of a female *P. guinanensis*. Both photos were taken by X. Xiao in May 2024.

The Guinan toad-headed lizard (*Phrynocephalus guinanensis*) presents a unique model for investigating pterin-based coloration in animals. As an endemic species inhabiting the eastern edge of the Qinghai–Tibetan Plateau, *P. guinanensis* is exclusively distributed in a desert dune environment (Mugetan Desert) spanning an area of 950 km^2^ [26,27] (Figure 1A,B). Adult lizards exhibit remarkable sexual dimorphism in ventrolateral coloration, with males displaying bright red (Figure 1C) and females displaying light green (Figure 1D). This sexually dimorphic pattern is unique to Mugetan and absent in its closely related species *P. putjatai* outside the desert [26]. The colors of adults hardly change during breeding and non-breeding seasons, nor do they change with age. Furthermore, Lu et al. [28] compared the skin chemical components between *P. guinanensis* and *P. putjatai*, identifying high concentrations of pterins but a low level of carotenoids in *P. guinanensis* males. This finding implied the involvement of pterins in producing their red coloration. However, the conclusion was not robust due to the small sample sizes (only three replicates) and the measurement of only a few specific pterin chemicals. In addition, the function of the red coloration remains unknown. Therefore, further studies on the biology of red coloration in *P. guinanensis* offer an excellent opportunity to investigate pterin-based coloration.

Here, we conducted an integrated investigation into the red coloration of males in *P. guinanensis*. Our objective was to address two fundamental questions: (1) Is the red coloration of *P. guinanensis* pigmentary, and if so, what are its chemical components? (2) Does the red coloration convey information about male quality? To answer these two questions, we employed a series of research approaches, including microscopy, metabolomics, spectrophotometry, visual modeling, and behavioral experiments. Our study aims to contribute a novel case to the understanding of pterin-based coloration in animals.

## 2. Materials and Methods

### 2.1. Study Site

We selected a study site in the southern Mugetan Desert (35.59° N, 100.92° E) to investigate the red coloration of males in wild populations. We sampled a total of 75 males during the breeding season in May 2024 for the following: spectral, body size, and performance measurements, and behavioral experiments. In addition, 28 females were also collected for behavioral experiments. All lizards were healthy adults, which were captured by noosing in the field and then housed individually in L:W:H = 20 cm × 20 cm × 10 cm cages indoors. They were released at their capture locations after all the work had finished.

### 2.2. Characterization of Red Color Production

The sexually dimorphic coloration of the ventrolateral region provided the opportunity to determine the characterization of red color production of males by comparing them to females. We first examined the ultrastructure of the dermal layer of ventrolateral skin using transmission electron microscopy (TEM). A total of 12 skin samples from 6 males with red color and 6 females with green color were used, originally sampled in 2020 from the same study site and have been utilized in a previous study [28]. TEM imaging was performed following the standard protocol (Supplementary Protocol S1) at the Bio-ultrastructure Analysis Lab of the Analysis Center of Agrobiology and Environmental Sciences, Zhejiang University, Hangzhou, China. To further characterize the chemical components responsible for the coloration, we conducted untargeted metabolomic identification of the skin samples using a Vanquish Liquid Chromatography (Thermo Fisher Scientific; Waltham, MA, USA) coupled to a Orbitrap Exploris 120 Mass Spectrometry System (LC-MS; Thermo Fisher Scientific; Waltham, MA, USA). Red-colored skin samples from 6 males served as the experimental group, while 6 non-red skin samples from females were used as the control group. We sliced 25 mg of skin from each sample, and LC-MS identification was carried out at Tsingke Biotech Co., Ltd., in Beijing, China.

### 2.3. Color Measurements and Visual Modeling

We quantified the spectral information of the ventrolateral coloration of 75 males using a Jaz optic spectrophotometer (Ocean Optics Inc.; Orlando, FL, USA) with a PX2 light source. Six surface locations were measured from both the left and right sides of the ventrolateral region for each individual (Supplementary Figure S1). Color data were normalized using a white 99% WS-1 standard (Labsphere; North Sutton, NH, USA) and a black card (RAL; Bonn, Germany). We conducted spectral analysis with the R package “pavo” version 2.9.0 to obtain color parameters—hue, chroma, and brightness—for each individual [29,30]. In addition, we photographed the abdomen of each lizard with a ColorChecker passport (Calibrite LLC; Wilmington, DE, USA) from a fixed angle using a digital camera (α6300, SONY; Kanagawa, Japan), and then four observers scored the degree of red coloration on an intensity scale from 1 to 9 (1 being light red, 9 being deep red). Furthermore, considering the color signal received through the visual system of lizards, we utilized visual modeling to calculate the chromatic (∆S) and luminance (∆L) contrasts of the red color against the sand dune background. Due to the lack of spectral sensitivity data of *P. guinanensis*, we used the visual system of the agamid *Ctenophorus ornatus* in this analysis [31,32]. The modeling followed the description by Qiu et al. [33], with λmax of UVS, SWS, MWS, and LWS set to 360, 440, 493, and 571 nm, respectively, and photoreceptor class densities of 1:1:3.5:6.

### 2.4. Body Size, Bite Force, and Testosterone Measurements

We measured snout–vent length (SVL), body mass (BM), tail length (TL), head length (HL), and head width (HW) to represent the body size of each individual. BM was measured to the nearest 0.01 g using a digital scale. Other lengths were measured to the nearest 0.01 mm using a vernier caliper (DL91200, Deli Group Co., Ltd.; Ningbo, China). We also collected in vivo bite force data, a common indicator of performance in lizards. Each individual was tested three times using a piezoelectric force transducer instrument (VXT500, 0–50 N, Viste; Shenzhen, China), with the largest value recorded as the bite force. Rectal temperature was measured concurrently with bite force using a digital thermometer (UT321, UNI-T; Dongguan, China). Additionally, to investigate the potential influence of sexual hormones on color expression, we measured the concentration of testosterone in the serum of males using competitive radioimmunoassay (RIA) at North Institute of Biotechnology Co., Ltd., Beijing, China [34]. Blood samples (50 µL) were collected from the tail base (1 cm from cloaca) using a lancet and a capillary tube after behavioral trials. Serum samples were then extracted by centrifugation (MC-10Pro, Joanlab; Huzhou, China) from the blood samples at 3000 rpm for 10 min.

### 2.5. Female Preference and Male–Male Competition Trials

The sexual dimorphism and intra-population variations of the coloration suggested its potential contribution in sexual selection. Thus, we conducted female preference and male–male competition experiments for males with light and deep red coloration during the breeding season (May 2024) in the field to investigate its role in sexual selection. The experimental setup consisted of three chambers at the sampling site. Two small chambers (L:W:H = 40 cm × 40 cm × 30 cm) housed individual males, separated by an opaque clapboard. A larger chamber (L:W:H = 80 cm × 40 cm × 30 cm) housed a female, allowing it to observe both males through a custom-made transparent glass clapboard (transmittance = 93.94%). Lizards were captured 48 h prior to each trial and fed two mealworms 24 h before the trial. Males were divided into two groups based on their red chroma and ∆S against sand substrate (light group: red chroma < 0.4, ∆S < 1; deep group: red chroma > 0.45, ∆S > 2.5). Body size was controlled to ensure SVL differences within each male pair would not exceed 5 mm. Females were randomly assigned to male pairs. All trials were conducted between 10:00 a.m. and 4:00 p.m. on sunny days.

In the female preference trials, two males and one female were initially placed in their respective chambers to acclimate for 15 min. During acclimation, the transparent layer was covered by a paper board. Subsequently, the paper board was removed, allowing visual contact between the female and males, although the males remained separated by the opaque clapboard. If, during the experiment, the female exhibited a positive response towards one male and attempted to approach the male for more than one minute, we considered this as the female’s preference. If the female displayed interest in both males, the first one to elicit a response was deemed the preferred male. If the female showed no preference within 30 min of removing the paper board, the trial was terminated.

Since we found that the males hardly competed with each other in the absence of females in the pilot trials, a female was kept in the formal trials to stimulate the males. Thus, in male–male competition trials, the lizards underwent the same pre-trial acclimation process as in the female preference trials, remaining in their respective chambers for 15 min. Subsequently, both the opaque clapboard separating the males and the paper board covering the transparent layer were removed, allowing the males to interact with each other in the presence of the female. A competition was deemed to have occurred if one male initiated an attack or chased away the other one, with the fleeing lizard considered the loser. The trial was terminated upon the conclusion of a competition to prevent injury. If no competition occurred within 30 min, the trial was also ended.

### 2.6. Statistical Analyses

All data in this study were analyzed using R version 4.2.2 (R Development Core Team, 2010). A two-sample Student’s *t*-test was employed to identify differentially expressed metabolites in the skin samples. Linear models were used to analyze the relationships between body size, bite force, testosterone level, and red coloration, with cloacal temperature included as a covariate for bite force. Red chroma, manual scores, ∆S, and ∆L were used as the representative color parameters. A one-sample Student’s *t*-test was used to assess the significance of SVL differences between male pairs in the behavioral trials. The significance level of the results of female preference and male–male competition in the behavioral experiments was evaluated using a binomial test.

## 3. Results

### 3.1. Composition of Red Coloration

**Figure 2:**
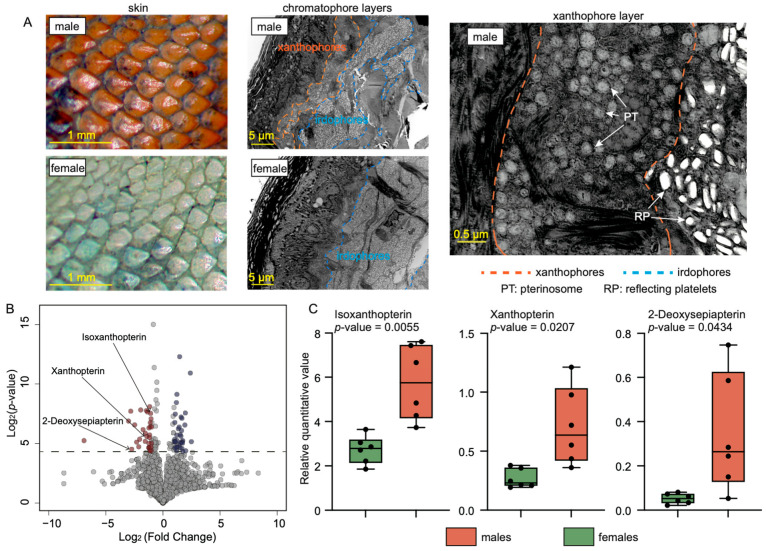
Characterization of red color production. (**A**) Microstructure of red and green skins from males and females, respectively. A xanthophore layer with pigment vesicles was identified in males’ skin. (**B**) Volcano plot for the expression of metabolites in untargeted metabolomic analysis. Red dots represent the significantly up-regulated metabolites in males, whereas blue dots represent the significantly up-regulated ones in females. (**C**) The relative expression of three pterin chemicals in males and females.

Using TEM, we observed a distinct difference in skin ultrastructure between males and females. Both male and female skins possessed an iridophore layer filled with reflecting platelets. In male skins, however, a prominent xanthophore layer containing numerous pigment vesicles was also observed. This layer was absent in female skins (Figure 2A). These findings strongly suggested that the pigments within the xanthophores are responsible for the sexually dimorphic red coloration in males.

We employed LC-MS to characterize the potential pigments in male skins. Through metabolomic identification, we detected a total of 2025 metabolites in the skin samples, of which 68 exhibited significant differentiation between the two sexes (fold change > 2; *p*-value < 0.05; Supplementary Table S1). Notably, 32 metabolites were specifically up-regulated in males (Figure 2B). Further analysis of these up-regulated metabolites revealed the presence of three pterin derivatives—isoxanthopterin (logFC = 1.08, *p*-value = 0.0055), xanthopterin (logFC = 1.41, *p*-value = 0.0207), and 2-deoxysepiapterin (logFC = 2.74, *p*-value = 0.0434) (Figure 2C). No carotenoid compounds were identified in either males or females. This provided strong evidence that the pigments within the xanthophores of males belong to the pterin family.

### 3.2. Color Variation in Wild Population

**Figure 3:**
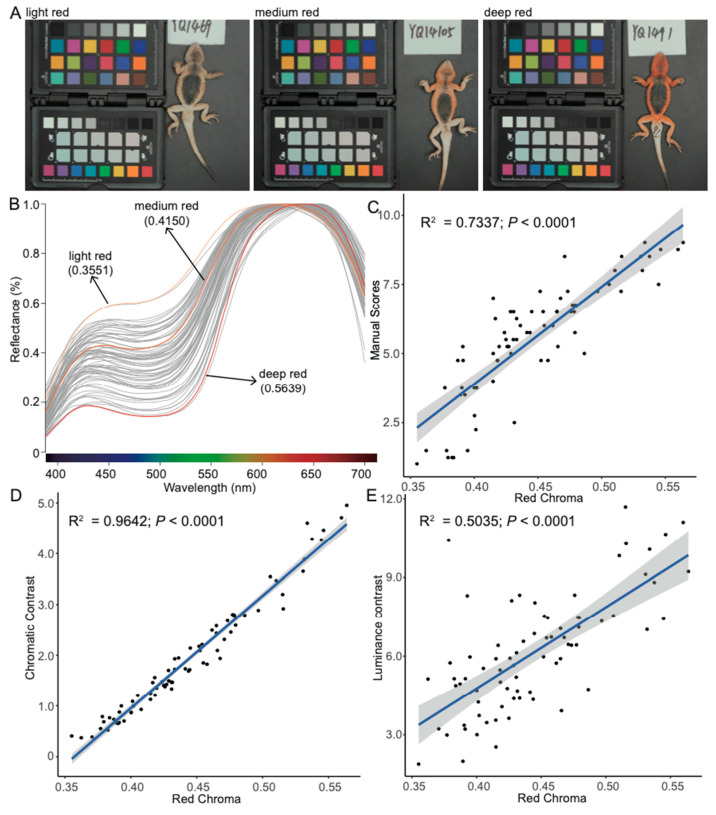
Red color measurements. (**A**) Photographs of ventrolateral region for three individuals represent light red, medium red, and deep red. (**B**) Normalized spectral curves of 75 males indicate the red color variation within the population. The scores in bracket give the red chroma of the three cases corresponding to the above photographs. (**C**–**E**) Linear models of relationships between red chroma and manual scores, chromatic contrast, and luminance contrast, respectively.

Through extensive sampling, we observed that the red coloration of males was continuous in wild population, varying from light to deep red (Figure 3A). Red chroma (S1R), the most commonly used metric for red intensity, ranged from 0.3551 (light red) to 0.5639 (deep red), indicating intraspecific color variation within the population. Normalized spectral curves revealed the major differences between individuals in the 400–600 nm wavelength range, with deeper red individuals exhibiting lower reflectance (Figure 3B). Normalized luminance (B2) between 400 and 600 nm reflected this pattern and was significantly correlated with red chroma (linear model: R^2^ = 0.9664, *p*-value < 2.2 × 10^−16^; Supplementary Figure S2). In addition, color variation was also assessed using manual scores (1–9 intensity scale) by four observers, and the average scores significantly correlated with red chroma (linear model: R^2^ = 0.7147, *p*-value < 2.2 × 10^−16^; Figure 3C). Also, using a lizard visual system model, we calculated chromatic (∆S) and luminance (∆L) contrasts of the skin color against the sand dune substrate. Both contrasts were significantly correlated with red chroma, especially the ∆S (linear model: R^2^_∆S_ = 0.9642, *p*-value < 2.2 × 10^−16^; R^2^_∆L_ = 0.5035, *p*-value = 1.03 × 10^−12^; Figure 3D,E).

### 3.3. Relationship between Body Size, Bite Force, Testosterone and Red Color

**Figure 4:**
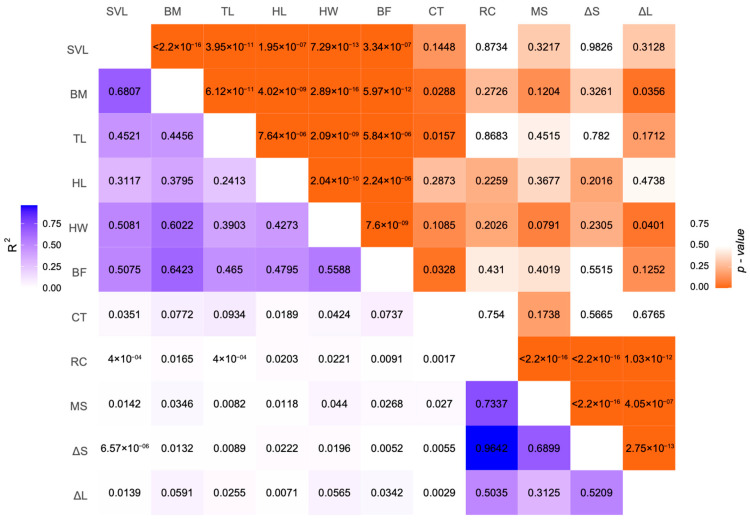
Relationships between morphological and performance traits and color parameters. The upper triangle denotes the *p*-values of the linear models; the bottom triangle denotes the R^2^ values of the same models. The color bars indicate the corresponding values. SVL: snout–vent length; BM: body mass; TL: tail length; HL: head length; HW: head width; BF: bite force; CT: concentration of testosterone; RC: red chroma; MS: manual scores; ∆S: chromatic contrast; ∆L: luminance contrast.

We obtained the data of seven traits for the lizards—SVL (mean 66.68; range 56.22–76.93 mm), BM (mean 11.45; range 6.90–16.57 g), TL (mean 68.47; range 58.43–77.29 mm), HL (mean 15.34; range 13.04–17.65 mm), HW (mean 15.12; range 12.77–17.73 mm), BF (mean 8.55; range 5.4–10.6 N), and CT (mean 0.045; range 0.008–0.019 ng/mL). Regarding body size, the five traits—SVL, BM, TL, HL, and HW—were significantly correlated with each other (linear model: all *p*-values < 0.0001). Bite force was significantly correlated with body size, particularly BM (linear model: R^2^ = 0.6423, *p*-value = 5.97 × 10^−12^) and HW (linear model: R^2^ = 0.5588, *p*-value = 7.06 × 10^−9^). Similarly, the concentration of testosterone in serum was significantly correlated with BM (linear model: R^2^ = 0.0772, *p*-value = 0.0288), TL (linear model: R^2^ = 0.0934, *p*-value = 0.0157), and bite force (linear model: R^2^ = 0.0737, *p*-value = 0.0328). For the relationship between color parameters and those traits, red chroma, manual scores, and ∆S were not correlated with body size, bite force, or testosterone level. ∆L was found significantly correlated with BM (linear model: R^2^ = 0.0591, *p*-value = 0.0356) and HW (linear model: R^2^ = 0.0565, *p*-value = 0.0401). All detailed information regarding these relationships are summarized in Figure 4.

### 3.4. Female Choice and Male–Male Competition Trials

**Figure 5:**
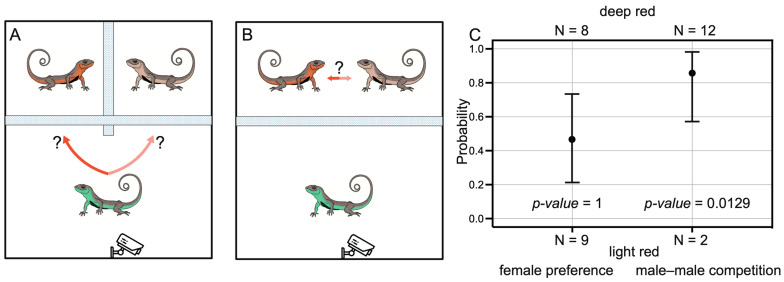
Behavioral trials on the function of red coloration in sexual selection. (**A**) Illustration of female preference trials. (**B**) Illustration of male–male competition trials. (**C**) Results of the two trials, where the probability and *p*-values were estimated from binomial test.

We conducted 24 female preference trials (Figure 5A), controlling for male SVL within each group (*p*-value = 0.2659 by Student’s *t*-test). Evidence of female preference was detected in 17 trials, with no significant preference observed between light red and deep red males (nine light red and eight deep red males favored by females, *p*-value = 1 by binomial test). Similarly, 23 male–male competition trials were conducted (Figure 5B), also controlling for SVL (*p*-value = 0.1821 by Student’s *t*-test). Evidence of competition was detected in 14 trials. Interestingly, deep red males exhibited significant dominance in competition, winning in 12 trials and only losing in 2 trials (*p*-value = 0.0129 by binomial test; Figure 5C).

## 4. Discussion

In this study, we conducted an integrated investigation into the red coloration of males in *P. guinanensis*. Using transmission electron microscopy and untargeted metabolomic identification, our results suggested that the red color was caused by pterin pigments within xanthophores. Through a series of approaches involving field sampling, color quantification, visual modeling, trait measurements, and behavioral experiments, we revealed that the red coloration was not correlated with body size, bite force, or testosterone level, nor did females exhibit preference bias toward the red intensity. However, males with deep red displayed a significant higher likelihood of winning in male–male competitions, which indicated that the pterin-based red coloration in *P. guinanensis* was probably a signal of male quality. Our results provided a novel case for the evolutionary mechanisms underlying the formation and maintenance of biological coloration.

Pterin-based red coloration in *P. guinanensis* appeared to convey information about male quality, suggesting that it may play a role in honest signaling. As another major source of conspicuous coloration, carotenoids cannot be synthesized endogenously by animals and are required to be acquired through dietary intake [35,36]. Thus, carotenoid-based colors are thought to be reliable indicators of individual quality, potentially reflecting an individual’s physiological condition or social rank for gaining resources, especially in resource-limited environments [37,38]. For instance, the plumage coloration of male house finches was determined by carotenoids, which reflected the nest attentiveness and overwinter survival, and was preferred by females [15]. However, as endogenously produced pigments, the role of pterins as honest signals remains unclear [18]. In the traditional view, only costly signals were thought to guarantee honesty, as deceivers could easily undermine the reliability of signals that were not costly to produce [39]. Thus, endogenously produced pigments were previously considered to have lower signal value because their precursors were less costly to obtain [36,40]. For pterins, the production costs are thought to be negligible, as they are synthesized de novo from the readily available guanosine triphosphate (GTP) [41,42]. Yet, it is now widely accepted that signals do not have to be costly to be honest, according to both theoretical and empirical studies [43,44]. In this study, we found that the red intensity of males significantly predicted the outcome of male–male competition during the breeding season. For this species, the potential advantage in competition is a crucial indicator of male quality, which can enhance fitness in terms of access to females and defending territories. Thus, our results indicated that the red coloration truthfully reflected the male quality of *P. guinanensis*, suggesting its potential role in honest signaling. Nevertheless, we found that the correlation between pterin-based red coloration and commonly used quality traits was weak. For example, the chromatic parameters (red chroma and ∆S) correlated with neither body size nor bite force. ∆L was found significantly correlated with body mass and head width, but this referred to only the perceived brightness compared to background, and the relationship was weak (both R^2^ < 0.06). Also, the red color was not regulated by the testosterone level, which was reasonable in this species since the red color also exists outside of the breeding season. All the evidence suggested that the mechanism of pterin-based coloration being signals could be complex, warranting further investigation.

Pterin-based red coloration potentially linked to a suite of internal traits associated with males’ fighting ability, implying its reliability as a signal. In fact, pterin metabolism is vital to multiple physiological processes, which may thus be associated with crucial functions for organisms [45,46]. For instance, in male water dragons, pterin-based red coloration was negatively associated with parasite resistance [47], whereas in pierid butterflies, nitrogen intake during the larval phase correlated with the intensity of leucopterin-based color in adult wings [48]. The core process of pterin biosynthesis converts GTP into tetrahydrobiopterin (BH4), an essential redox cofactor promoting cell proliferation, tyrosine synthesis, and H_2_O_2_ scavenging [49,50]. Thus, pterin and derivative deficiencies are linked to severe health and developmental disorders, including neurological disease and impaired growth [51]. Here, our results suggested the significant role of red coloration in sexual selection, similar to the other pterin-based colors in species such as cabbage butterfly [48,52] (*Pieris brassicae*) and bluefin killifish [46] (*Lucania goodei*). However, male lizards with deep red exhibited more aggressive and dominant behavior in competition with other males, rather than the color favored by females. This phenomenon was also found in other lizard species. For instance, male common wall lizards with green dorsal coloration dominate in courtship without female preference [53,54]. Male–male competition and coercive mating are prevalent in lizards [55,56,57]). It is plausible that pterins producing red colors also participate in other metabolic processes, which can enhance the fighting ability for the males. The association between pterins and metabolism makes it difficult to forge pterin signals, as individuals cannot easily change their internal physiological state, providing the possibility that pterin-based red coloration could be a reliable signal against deception in *P. guinanensis*. Similarly, further studies are indeed required to elucidate the exact physiological and metabolic differences between males with varied red intensity.

Why animals utilize pterins as a major source of conspicuous coloration remains a controversial question [18]. Although carotenoids, which can produce similar hues, often exhibit a clearer correlation with individual quality in many species, pterins are still favored by numerous organisms across the evolutionary spectrum [12,13]. One potential explanation posits that pterins may compensate for the lower environmental availability of carotenoids. For instance, in agamid lizards, habitats with reduced productivity are associated with lower carotenoid concentrations but higher pterin concentrations [58]. In the case of *P. guinanensis*, a species primarily inhabiting the sand dune habitats in the Mugetan Desert, the scarcity of carotenoids in the environment may have led to the utilization of pterins as the primary source of red coloration. However, the compensation of carotenoids by pterins is not a straightforward process [59,60]. A more comprehensive understanding of the molecular pathways involved in generating pterin-based coloration is necessary to further illuminate the relationship between color and its signaling information.

## 5. Conclusions

In this study, we conducted an integrated investigation into the red coloration exhibited by males of *P. guinanensis*. Through transmission electron microscopy and untargeted metabolomic identification, our results suggested that this coloration was attributable to pterin pigments localized within xanthophores. Combining a series of approaches encompassing field sampling, color quantification, visual modeling, trait measurements, and behavioral experiments, we identified that the red coloration was not correlated with body size, bite force, or testosterone level, nor did females show preference bias to it. Notably, however, males exhibiting a deeper red coloration demonstrated a significantly higher probability of success in intrasexual competition, suggesting that the pterin-based red coloration in *P. guinanensis* serves as a signal of male quality, potentially linked toward fighting ability through internal metabolic processes. Our results contributed a novel perspective to the understanding of the evolutionary mechanisms underpinning the formation and persistence of biological coloration.

## Data Availability

The raw data and R scripts for color/trait analyses used in this study are available in Science data bank (ScienceDB) at http://doi.org/10.57760/sciencedb.12374.

## Supplementary Materials

The following supporting information can be downloaded at https://www.mdpi.com/article/10.3390/ani14202923/s1, Figure S1: Illustration of the ventrolateral region for spectral sampling; Figure S2: The linear relationship between red chroma and normalized brightness; Table S1: Differentially expressed metabolites between male and female skins; Protocol S1: Sample processing procedures of TEM.
