# Supplementary material for: Pterin-Based Red Coloration Predicts the Outcome of Male–Male Competition in Guinan Toad-Headed Lizard
Source: Animals (Basel). 2024 Oct 11;14(20):2923. doi: 10.3390/ani14202923 (PMC11503834; doi:10.3390/ani14202923)
Supplement: Supplementary file 1 [file animals-14-02923-s001.zip › animals-3202657-supplementary.pdf]

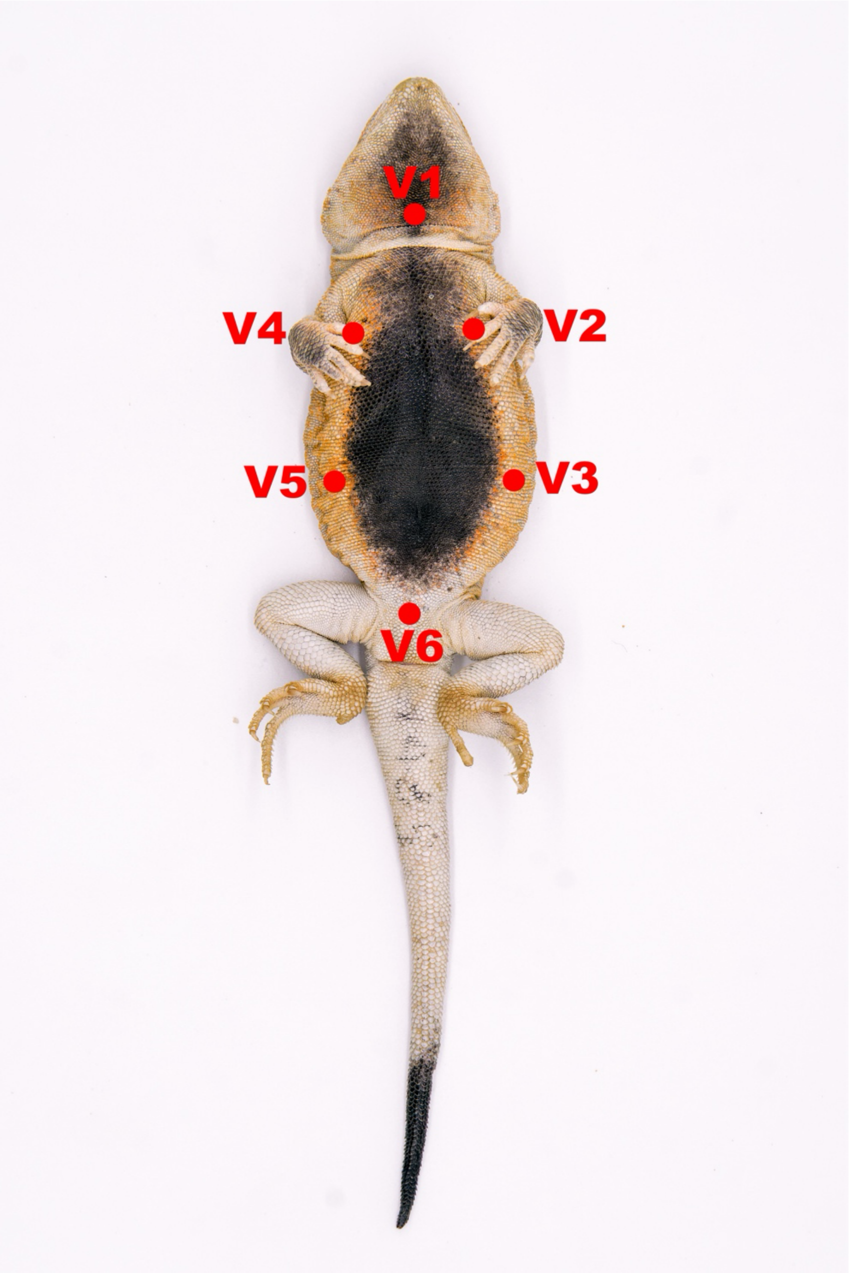

Figure S1 Illustration of the ventrolateral region for spectral sampling. The red spots indicate the sampling sites to collect spectral data.

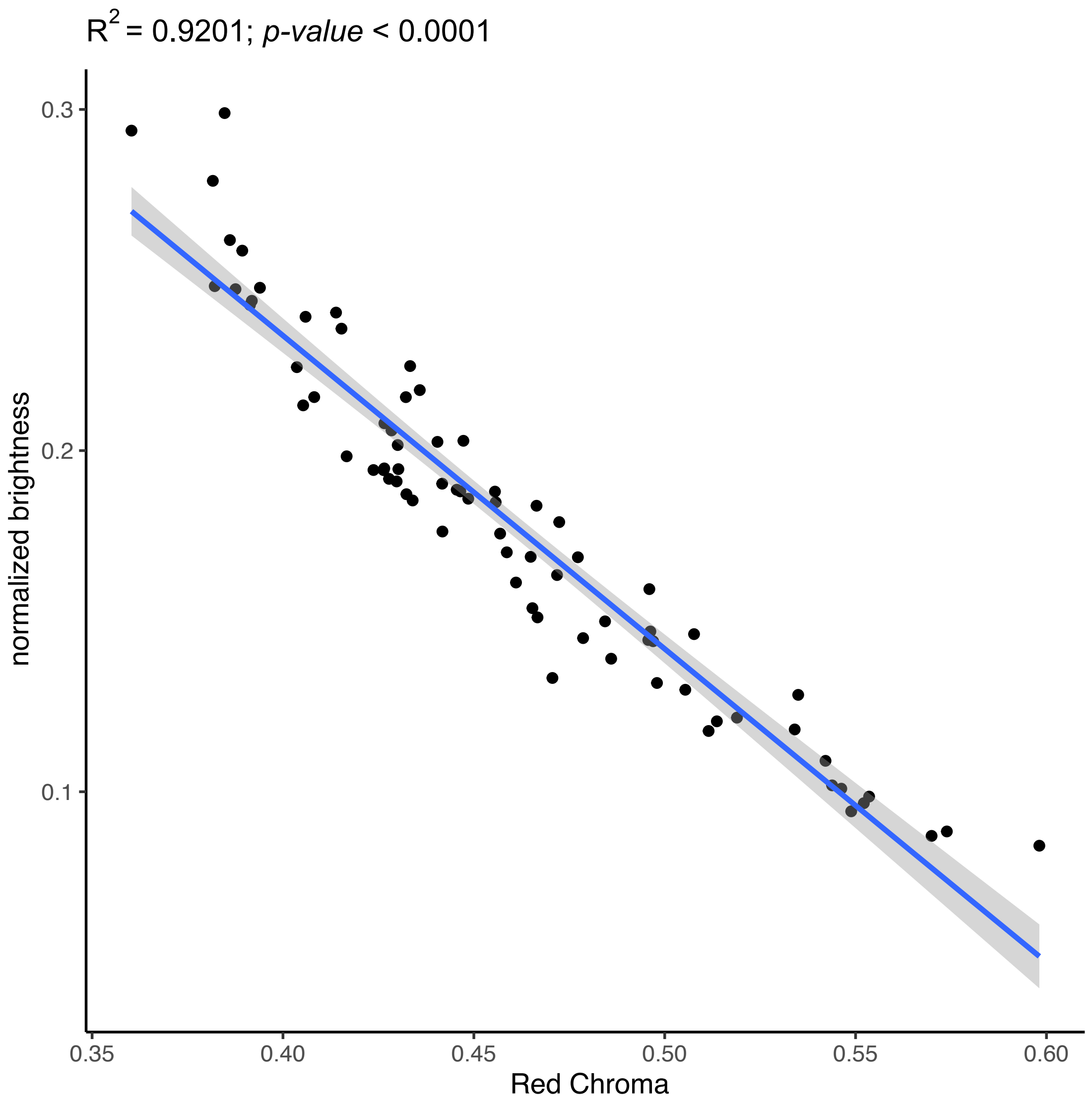

Figure S2 The linear relationship between Red Chroma and normalized brightness.

Table S1 Differentially expressed metabolites between male and female skins

metabolites	relative quantity (females)	relative quantity (males)	fold change	*p-value*
Upregulated metabolites in male skins				
Pipecolic acid	0.00374989	0.008801092	2.347026624	0.03984445
Isoxanthopterin	2.72603877	5.757072083	2.111881954	0.0054514
Lactate	58.5895694	131.0815543	2.237284822	0.00366883
Hygric acid	0.00374989	0.008801092	2.347026624	0.03984445
Acadesine	0.018311917	0.03938627	2.150854589	0.01179187
Nipecotic acid	0.00374989	0.008801092	2.347026624	0.03984445
4-Amino-9-methoxypsoralen	0.002588122	0.00518157	2.002058121	0.01200584
Xanthopterin	0.266390115	0.709499302	2.663384494	0.02072987
6-Amino-3-methyl-3H-naphtho[1,2,3-de]quinoline-2,7-dione	0.006827818	0.018695262	2.7381018	0.00462724
2-Deoxysepiapterin	0.051612168	0.344287877	6.670672591	0.043419
LPE(P-20:0)	0.02840005	0.057532022	2.025771844	0.04767424
Ethyl 5-(2-chloroacetyl)-2,4-dimethyl-1H-pyrrole-3-carboxylate	0.00585162	0.012057553	2.060549614	0.00745172
Damnacanthal	0.007825937	0.015674418	2.002880805	0.03063418
D-Glucosaminic acid	0.04592627	0.15417902	3.357098671	0.01392218
4-(4-tert-Butylphenyl)-1,3-thiazol-2-ylamine	0.000963042	0.003964442	4.116583741	0.02442471
Methyl paraoxon	0.002369158	0.005092443	2.149473618	0.00935547
Campestanol	0.008746932	0.018055413	2.064199656	0.03854184
[2-(1H-Tetraazol-5-yl)phenoxy]acetic acid	0.002449725	0.019847453	8.101910759	0.00848802
Rabeprazole sulfone	0.000793743	0.003454715	4.352433406	0.03774601
1-Ethyl-5-methyl[1,2,4]triazolo[4,3-a]quinolin-8-yl methyl ether	0.00202458	0.011293792	5.578338059	0.01060441
3-Amino-4,4,4-trifluorobutanoic acid	0.004170247	0.009392133	2.25217693	0.01901053
2-Hydroxy-3-(trifluoromethoxy)benzoic acid	0.050332077	0.14710837	2.92275582	0.01719307
N-Acryloyl-DL-aspartic acid	0.448740433	1.149346018	2.561271356	0.00499183
Dulxanthone_F	0.003760223	0.008285803	2.203540215	0.04801023
1,3-Dihydroxyacetone dimer	0.030857915	0.11905121	3.858044524	0.00445203
Clasto-lactacystin .beta.-lactone	0.010953332	0.032157798	2.935891956	0.02760445
3-Hydroxy-5-methoxy-3-(methoxycarbonyl)-5-oxopentanoic acid	0.03816564	0.269964575	7.073497916	0.00479958
2-(2-Naphthyl)-1,3-benzoxazol-5-amine	0.002490357	0.00581731	2.335934478	0.01269339

Cephamycin C	0.002563753	0.005792688	2.259456188	0.02218908
Ginsenoyne_A	4.02267E-05	0.00486057	120.8295492	0.02634181
Bupropion	0.0022443	0.005242842	2.336069896	0.0309505
Pentadecanoic acid	0.014606347	0.072038293	4.93198573	0.02869199
Upregulated metabolites in female skins				
LPC(16:0)	8.622656662	3.700719558	2.32999462	0.02620266
Thiamine	0.090567557	0.02796922	3.238115209	0.028508472
LPC(18:2/0:0)	1.130930675	0.479324463	2.359426154	0.038682189
6-(Trifluoromethyl)-1H-pyrazolo[3,4-d]pyrimidin-4-ol	0.08643268	0.042640495	2.027009302	0.024806912
Myricetin	0.008452657	0.003332298	2.53658461	0.013273623
4-Chloro-5,6,7,8-tetrahydroquinazolin-2-amine	0.699450842	0.304225658	2.299118508	0.046956737
5-(3-Chlorophenyl)-5-methylimidazolidine-2,4-dione	1.245722965	0.605430812	2.05758105	0.00921545
N-Methyl-N-(methylsulfonyl)glycine	6.763245035	2.909211522	2.324769095	0.019403291
N-Acetyl-L-Prolinamide	0.012227975	0.006029145	2.028144123	0.028371825
5-Hydroxy-6E,8Z,11Z,14Z-eicosatetraenoic acid, 1,5-lactone	0.061199873	0.022284362	2.746314848	0.000199177
Flunitrazepam	0.007594548	0.002293812	3.310885738	0.026382678
2,2,4,7-Tetramethyl-1,2-dihydroquinoline	0.06715407	0.021667823	3.099253163	0.014802179
3-Sulfinoalanine	1.588435948	0.751832408	2.112752697	0.00565802
1H-Pyrazol-3-amine	0.046261908	0.015569183	2.971376683	0.028950774
2-Naphthalenethiol	0.435231028	0.153549725	2.834463092	0.018690548
N-(4-Chloro-3-nitrophenyl)-3-methylbenzamide	0.103809203	0.034512185	3.00790006	0.006739502
N-(3-Chloro-4-methylphenyl)-4-nitrobenzamide	0.028364077	0.010723297	2.645089243	0.034070131
1-(3-Chlorophenyl)-3-(4-sulfamoylphenyl)urea	0.018772287	0.006496162	2.889750537	0.031000285
3',4',6-Trihydroxy-3,5,7-trimethoxyflavone	0.001092008	0.000386723	2.823745658	0.04013051
N-(2-Chlorobenzyl)-1-propanamine	0.023139015	0.007326805	3.158131682	0.007243969
Pyridine	0.006229898	0.002096697	2.971292144	0.020372416
2'-Hydroxy-4'-methoxyacetophenone	0.843718183	0.362185553	2.329519153	0.001797936
Diflunisal	0.111066908	0.02108565	5.267416861	0.000518882
N-(2-Hydroxyethyl)-2-(3-nitro-1H-1,2,4-triazol-1-yl)acetamide	0.037925657	0.006758165	5.61182757	0.027971003
LysoPC(20:4(5Z,8Z,11Z,14Z))	0.296929352	0.093494218	3.175911377	0.041663847
LysoPC(18:3(6Z,9Z,12Z))	0.257050938	0.12192463	2.10827737	0.028413711
hydroxypropyl hexopyranoside	0.034411025	0.014988595	2.295813917	0.038239684

N-Diphenylmethanesulfonamide	0.287255378	0.127154607	2.259103196	0.002891013
N-Nonanoyl-L-homoserine lactone	0.045383043	0.013862368	3.273830434	0.030680297
Homovanillyl alcohol-4'-glycoside	0.394084543	0.147523767	2.671329185	0.012028983
2-[(Dibenzylamino)carbonyl]benzoic acid	0.004903133	0.001469002	3.337731634	0.049707743
Thioguanine	0.025137472	0.012139693	2.07068424	0.040287476
2-[(2-Fluorophenyl)amino]acetohydrazide	0.1619793	0.042892022	3.776443583	0.010694071
methyl ester	0.264486222	0.068106197	3.883438433	0.005321202
1H-1,3-Benzodiazole-5-carbaldehyde	0.126252467	0.031698475	3.982919262	0.047746593
M192T132	0.070424268	0.033492823	2.102667417	0.02830906

Protocol S1 Sample processing procedures of TEM

1.Double fixation: The sample was first fixed with 2.5% glutaraldehyde in phosphate buffer (0.1M， pH7.0) for more than 4h; washed three times in the phosphate buffer(0.1M， pH7.0) for 15min at each step; then postfixed with 1% OsO_4 in phosphate buffer for 1-2h and washed three times in the phosphate buffer(0.1M， pH7.0) for 15min at each step.

Pay attention: 1. Wash with phosphate buffer completely

2. OsO_4 is very dangerous and toxic reagent, the using must be Carried out under teacher's guidance and must be in fume hood.

2.Dehydration: The sample was first dehydrated by a graded series of ethanol (30%， 50%, 70%, 80%) for about 15min at each step, then dehydrated by a graded series of acetone(90%,95%)for about 15min at each step. In the end, dehydrated twice by absolute acetone for 20 min respectively.

Pay attention: 1.After 50% ethanol, sample's tube must be covered.

2.Don't let sample out of solution for more than 1min!!!

3. Infiltration: The specimen was placed in 1:1 mixture of absolute acetone and the final Spurr resin mixture for 1h at room temperature, then transferred to 1:3 mixture of absolute acetone and the final resin mixture for 3h and to final Spurr resin mixture for **overnight**.

Pay attention: Keep drying!

4.Embedding, ultrathin sectioning, staining and observation: Specimen was placed in eppendorf contained Spurr resin and heated at 70℃ for more than 9h. The specimen was sectioned in LEICA EM UC7 ultratome and sections were stained by uranyl acetate and alkaline lead citrate for 5 to 10min respectively and observed in Hitachi Model H-7650 TEM.
